# Navigating family-witnessed resuscitation in hospitals: content analysis of free-text survey data

**DOI:** 10.1016/j.ijnsa.2025.100347

**Published:** 2025-05-15

**Authors:** Annette Waldemar, Ingela Thylén

**Affiliations:** a Department of Cardiology in Norrköping, and Department of Health, Medicine and Caring Sciences, Linköping University, SE, 581 83 Linköping, Sweden; b Department of Cardiology in Linköping, and Department of Health, Medicine and Caring Sciences, Linköping University, SE, 581 83 Linköping, Sweden

**Keywords:** Family-witnessed resuscitation, In-hospital cardiac arrest, Healthcare professionals, Attitudes, Barriers, Qualitative, Free-text responses

## Abstract

**Background:**

Although international guidelines advocate family-witnessed resuscitation, its implementation varies considerably across countries. Clinical practice is often shaped by healthcare professionals’ personal beliefs and preferences, rather than standardised practice or evidence-based protocols. While patients and families generally support family presence for its reassurance and emotional closure, healthcare professionals express concerns about its potential to cause trauma. Previous researchers have primarily examined nurses' perspectives, with limited focus on physicians’ experiences. Moreover, most studies have been conducted in emergency and intensive care settings, leaving a gap in understanding collaborative perspectives across diverse clinical settings.

**Objective:**

To describe nurses’ and physicians’ attitudes and experiences regarding family-witnessed resuscitation during adult cardiac arrest across various hospital departments and levels of care and to suggest key areas for targeted improvements and practical clinical applications.

**Design:**

A qualitative design was applied to analyse free-text responses from a questionnaire distributed to healthcare professionals participating in an educational intervention.

**Setting:**

Conducted in six Swedish hospitals, we included healthcare professionals from emergency care, internal medicine, cardiology, infectious diseases, and orthopaedic, thoracic, and abdominal surgery departments.

**Participants:**

A total of 96 nurses and 48 physicians, with a mean age of 42 years and 15 years of working experience, participated, of which 51 % had prior experience with family-witnessed resuscitation.

**Methods:**

A summative content analysis was conducted. Texts were repeatedly reviewed, condensed into keywords, coded, and grouped into sub-categories and categories, which were compared for similarities and differences. Keyword frequencies were calculated to summarise attitudes and experiences within each sub-category.

**Results:**

Three categories and seven sub-categories emerged. *Taking a stand or being indecisive* reflected the tension between balancing patient and family wishes and healthcare professionals’ preferences, with indecisiveness more common in cases involving vulnerable family members. *Working under pressure* encompassed challenges related to operational constraints during family-witnessed resuscitation, such as limited room capacity, confidentiality risks, insufficient family support, and disruptive family behaviours that could impair focus. *Helping or harming the family* captured the dual perception that family presence could facilitate understanding of the resuscitation process and support grieving if the patient did not survive, while also posing a risk of trauma for family members.

**Conclusions:**

Family-witnessed resuscitation presents significant ethical and practical challenges for healthcare professionals. Targeted training may be essential to prepare them for managing the cognitive, psychological, cultural, and familial complexities inherent in these situations. The development and implementation of clear, evidence-based local guidelines can support resuscitation teams in adopting a consistent, safe approach to family-witnessed resuscitation. Such initiatives can enhance healthcare practice by balancing patient and family needs with ethical and professional standards.

**Study registration:**

Not registered.

**What is already known**

• Despite endorsements from international organisations, the implementation of family-witnessed resuscitation policies remains limited in many countries and hospitals, with structured protocols, simulation-based training, and designated family support being essential for successful integration.
• Nurses generally support family-witnessed resuscitation for its psychological benefits to families, while physicians are more doubtful and express concerns about the risk of family trauma, staff stress, litigation, and logistical challenges during resuscitation efforts.
• Family-witnessed resuscitation can aid the grieving process and reduce post-traumatic stress symptoms in families without negatively affecting survival rates or resuscitation quality.

Alt-text: Unlabelled box

**What this paper adds**

• The implementation of family-witnessed resuscitation was highly variable, with healthcare professionals often making decisions based on personal values rather than evidence-based guidelines or policy.
• Healthcare managers might consider equipping staff with tools to address concerns about potential threats or violence, especially when meeting family members with criminal backgrounds, to enable healthcare professionals to navigate complex resuscitation situations and ethical challenges.
• Vulnerable family members, particularly children and adolescents with mental illness or cognitive impairments, may pose significant ethical challenges in preparing them for the emotional impact of witnessing resuscitation, which may be too traumatic for them to process.

Alt-text: Unlabelled box

## 1 Background

Family-witnessed resuscitation in hospitals has been a topic of clinical debate for over 40 years (Toy, 2023). While widely practiced in some countries, its implementation remains inconsistent globally and is more common in paediatric or out-of-hospital settings than in adult in-hospital care (Meghani, 2021; Tíscar-González et al., 2020). In Swedish hospitals, family-witnessed resuscitation is still uncommon, and opportunities for family presence during resuscitation remain limited (Waldemar and Thylén, 2021).

Family-witnessed resuscitation offers families the opportunity to witness resuscitation efforts, providing reassurance and reducing feelings of exclusion. It allows them to engage meaningfully by offering comfort, praying, or saying final goodbyes, which can support the grieving process and bring closure (De Stefano et al., 2016; Powers et al., 2023). Since the first study in the 1980s highlighting these benefits (Doyle et al., 1987), researchers have consistently shown the positive impact on families, who themselves express a strong desire to be present during resuscitation (Tíscar-González et al., 2020; Vardanjani et al., 2021).

Despite its recognised benefits and the existence of guidelines (Mancini et al., 2015; Mentzelopoulos et al., 2021), family-witnessed resuscitation remains a controversial practice in adult care (Johnson, 2017). The healthcare team decides whether to invite the family or respond to requests from the family to witness the resuscitation (Considine et al., 2022). Nurses tend to support the practice, while physicians are more hesitant, citing concerns about interference, litigation, or their own increased stress during resuscitation (Tennyson, 2019; Waldemar and Thylen, 2019). Healthcare professionals worry that family presence may be traumatic for unprepared family members and could hinder team performance (Afzali Rubin et al., 2020; Tíscar-González et al., 2021). Practical challenges, such as overcrowded rooms and reduced self-confidence during procedures, also contribute to reservations (Barreto et al., 2019; Waldemar and Thylen, 2019). However, researchers have shown that family-witnessed resuscitation does not negatively impact key procedural metrics, including time to defibrillation, quality of resuscitation, or patient outcomes (Goldberger et al., 2015; Oczkowski et al., 2015; Waldemar et al., 2021).

The principles of person-centred and family-centred care have gained increasing attention in recent years, emphasising the involvement of families in healthcare decisions and patient care environments (Clay and Parsh, 2016). Family-witnessed resuscitation aligns closely with these principles by addressing families’ emotional and relational needs during critical moments. It demonstrates the potential of integrating person-centred care into resuscitation practices, despite the ethical and practical tensions that persist (Bradford et al., 2024).

Training can improve healthcare professionals’ confidence and ability to support families during and after resuscitation, ensuring the practice is carried out safely and effectively (Grimes, 2020). To enable tailored training on family-witnessed resuscitation, it is crucial to address the persistent barriers to its implementation. A structured nationwide approach requires a comprehensive understanding of challenges spanning diverse clinical settings and professional roles. While most researchers on family-witnessed resuscitation focus on isolated contexts or specific professional groups, there is a significant gap in understanding the collaborative dynamics of healthcare teams across varying hospital environments. We addressed this gap by qualitatively analysing free-text survey responses from nurses and physicians who were participating in an educational intervention in Sweden.

### 1.1 Aim of the study

To describe nurses’ and physicians’ attitudes and experiences regarding family-witnessed resuscitation during adult cardiac arrest across various hospital departments and levels of care and to suggest key areas for targeted improvements and practical clinical applications.

## 2 Methods

### 2.1 Study design

We adopted a qualitative design to analyse free-text responses from a questionnaire addressing experiences and attitudes towards family-witnessed resuscitation. This questionnaire was originally developed by Fulbrook et al. (2005) and later translated and validated in Swedish (Axelsson et al., 2010). The questionnaire was distributed to healthcare professionals participating in a multicentre educational intervention conducted in 2022–2023. Quantitative data and outcomes from the intervention were reported elsewhere (Waldemar et al., 2024).

Methods are reported in accordance with Standards for Reporting Qualitative Research guidelines.

### 2.2 Sample

The study was conducted in one university hospital, one county hospital, and four district hospitals in Sweden. Study participants were nurses and physicians working in departments of emergency care; internal medicine; cardiology; infectious diseases; and orthopaedic, thoracic, and abdominal surgery. Of the 193 healthcare professionals who participated in the educational intervention study, 75 % (*n* = 144) provided free-text responses in the survey and were therefore included in this qualitative analysis.

### 2.3 Data collection

Background characteristics included data on sex, age, workplace, and years of professional experience. Questions related to experiences of family-witnessed resuscitation addressed whether the participant’s workplace had local protocols on family-witnessed resuscitation (yes/no), prior in-hospital experience with family-witnessed resuscitation (yes/no), and, if so, whether this experience was perceived as positive, negative, or both. Additional questions explored whether the participant had previously offered family members the opportunity to be present during resuscitation (yes/no) and whether they had been asked by a family member to allow presence during resuscitation (yes/no). The qualitative analysis focused on responses to three open-ended questions, with no word limit:1) Participants’ opinions, thoughts, experiences, or narratives about family-witnessed cardiac arrest in hospital settings; 2) The main reasons for not offering family members the opportunity to be present during a relative's resuscitation; and 3) The main reasons for offering family members the opportunity to be present during a relative's resuscitation.

### 2.4 Data analysis

#### 2.2.1 Summative content analysis

A modified summative content analysis inspired by Hsieh and Shannon (2005) was performed to analyse the free-text responses, focusing both on how healthcare professionals described their experiences and attitudes towards family-witnessed resuscitation and on the frequency of these experiences and attitudes. This type of analysis includes quantification of terms and content within the text, as well as interpretation of the underlying meaning, which can be used for open-ended qualitative survey responses to identify patterns in text through subjective interpretation (Hsieh and Shannon, 2005). The initial analysis was developed using an inductive approach with one of the researchers (AW, a specialist nurse with a PhD and over 30 years of experience in cardiac care) reviewing 14,838 words corresponding to approximately 28 A-4 pages with written text. The text was read several times and condensed into keywords: e.g., *The family interferes affects the work of the resuscitation team and takes the attention away from the patient*. These keywords were then shortened and coded: e.g., *stress, teamwork, interfering with resuscitation*. Codes were further grouped and sorted into preliminary sub-categories and categories that were compared based on differences and similarities. Some overlapping emerged, and the categorization was further revised and checked by the other member of the research team (IT, a registered nurse and associate professor with extensive experience in cardiac care and conducting qualitative studies). Finally, all keywords was calculated and the frequencies (%) of attitudes and experiences were counted and summarised under each sub-category. The Swedish quotations were translated verbatim into English by the second author (IT), ensuring the original meaning and tone were preserved.

### 2.5 Ethical considerations

An advisory statement was obtained from the Swedish Ethical Review Authority in Sweden (2022–01,974–01). The heads of the departments gave their written consent for participation at the department level and provided e-mail addresses for all nurses and physicians working with direct patient care within their department. Participation in the study was voluntary, and before answering the survey, the participants gave their consent digitally.

## 3 Findings

### 3.1 Demographics and experiences of family-witnessed resuscitation

A total of 96 nurses and 48 physicians with a mean age of 42.2 ± 10.4 years were included in this qualitative analysis of free-text data. As shown in Table 1, participants were experienced healthcare professionals, most commonly working in emergency, cardiology, or medical departments. While a quarter of participants reported exclusively positive experiences with family members present during resuscitation, about half had no prior experience of such situations.

**Table 1:** Socio-demographic characteristics.

Socio-demographic characteristics	Total
	*N* = 144
	*n* (%) 1
Sex	
Female	104 (72.2)
Male	40 (27.8)
Age (mean, ± standard deviation)	42.2 ± 10.4
Department	
Emergency	45 (31.3)
Cardiology	10 (6.8)
Medical	39 (27.1)
Surgical	9 (6.3)
Outpatient clinic	34 (23.6)
Other	7 (4.9)
Years of professional experience (mean ± standard deviation)	15.1 ± 9.7
Workplace having local protocols regarding family-witnessed resuscitation (yes)	27 (18.8)
Experiences 2	
No previous experiences of family-witnessed resuscitation (yes)	71 (49.3)
Previous positive experiences of family-witnessed resuscitation (yes)	37 (25.7)
Previous negative experiences of family-witnessed resuscitation (yes)	17 (11.8)
Previous negative and positive experiences of family-witnessed resuscitation (yes)	
19 (13.2)	
Previous experiences of offering family-witnessed resuscitation (yes)	46 (31.9)
Previous experiences of being asked by a family member to be present during cardiopulmonary resuscitation (yes)	21 (14.6)

1 Some missing values, which explains the difference in %.

2 Percent that answered yes to each statement.

Regarding institutional protocols, about a fifth reported that local guidelines on family-witnessed resuscitation were in place at their workplace. Of these, the vast majority (*n* = 25, 92.6 %) were employed in the emergency, cardiology, or medical departments.

### 3.2 Categories and sub-categories

In total, three categories and seven sub-categories emerged (Table 2).

**Table 2:** Categories and sub-categories.

Categories	Sub-categories
Taking a stand or being indecisive	Respecting patient and family wishes Challenges posed by vulnerable family members
Working under pressure	Navigating operational constraints
	Limited family support
	Disruptions from family behaviour and impaired focus
Helping or harming the family	Family presence facilitates understanding and grieving Traumatic sensory impressions

**Fig. 1:**
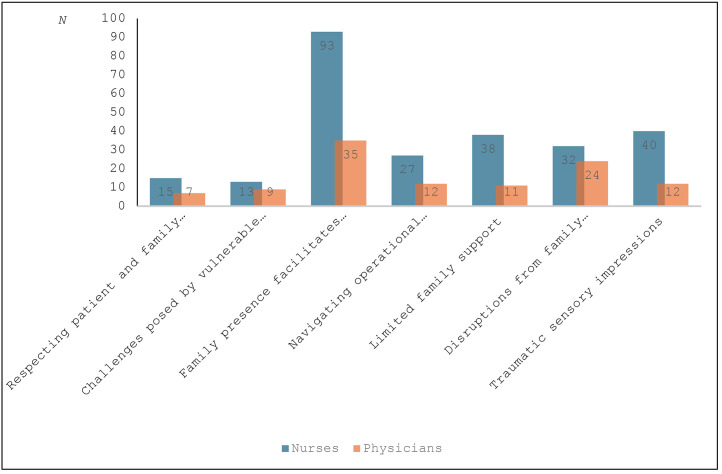
Proportions (%) of nurses’ and physicians’ attitudes and experiences generating free-text, divided into each sub-category (*N* = 144).

The proportions (%) of nurses’ and physicians’ attitudes and experiences generating free-text were divided into each sub-category as described in Fig. 1. Most participants provided free-text responses encompassing several sub-categories, capturing a wide range of perspectives and experiences on family-witnessed resuscitation in the hospital. Most free-text responses pertained to the sub-category *Presence makes the resuscitation understandable and facilitates the grieving process*.

#### 3.2.1 Taking a stand or being indecisive

Taking a stand or being indecisive encompassed experiences and attitudes regarding the importance of respecting both patient and family wishes concerning family-witnessed resuscitation, while balancing these with the healthcare professionals’ own preferences. Indecisiveness was particularly prominent in situations involving vulnerable family members. Of the three identified categories, 12 % of the reported experiences and attitudes were related to this first category.

##### 3.2.1.1 Respecting patient and family wishes

The participants highlighted that family-witnessed resuscitation was challenging since it is a relatively uncommon practice in hospital settings. This contrasts with out-of-hospital scenarios, where family involvement is more natural. As one nurse expressed, ‘Often families have started cardiopulmonary resuscitation at home before the ambulance arrives, so I believe it’s obvious that they should be present in the emergency room. I think that many physicians and nurses who don’t have prehospital experience forget this’.

Many participants emphasised the importance of offering family members the opportunity to be present during resuscitation. However, they expressed that patients’ wishes regarding family-witnessed resuscitation were rarely documented in the medical records beforehand, adding complexity to these situations. Participants also described instances where patients had wished for their family’s presence during resuscitation, but the family member became overwhelmed, disrupting the process. These situations highlighted the complexity of family dynamics in family-witnessed resuscitation decisions. Despite these challenges, participants recognised significant benefits of family presence during resuscitation. Family-witnessed resuscitation was seen as emotionally supportive for patients and as an aid to family members in processing the event. One nurse commented, ‘If the patient wakes up, it is advantageous if family members are present for the sake of the patient and the family’.

Witnessing the resuscitation was also noted to provide comfort and closure for family members, allowing them to recount the event to the patient during recovery or find peace if the patient does not survive. A physician reflected, *‘We don’t know what patients truly experiences during resuscitation, but hearing and knowing their family is present in their final moments must be comforting. For example, recent research on electroencephalograms in cardiopulmonary resuscitation suggests that dying individuals might perceive more than we assume’.* Overall, participants expressed mostly positive attitudes toward family-witnessed resuscitation, with many advocating for family inclusion.

Finally, participants highlighted the ethical importance of respecting family members’ preferences and ensuring they were present out of genuine desire rather than obligation. Working in a person-centred manner and showing respect were considered essential in family-witnessed resuscitation. Allowing family to be with their dying loved ones was viewed as providing peace and meaning, while denying this opportunity was seen as ethically problematic. As described by this physician, *‘It is obvious to me that family members should be involved. I have long and wide experience of paediatric cardiac arrests, suicides, traumatic, medical, geriatric, unexplained cardiac arrests – and my experience is the importance of giving family members the opportunity to stay. Family members are always there when I resuscitate if they want to be’*.

##### 3.2.1.2 Challenges posed by vulnerable family members

Family members with vulnerabilities, such as mental illness, cognitive impairments (e.g., dementia), severe autism, or Down syndrome, were perceived as potential barriers to family-witnessed resuscitation. Participants expressed concerns that such individuals might struggle to comprehend the situation, disrupt the resuscitation process, or experience exacerbated trauma. As one nurse described, *‘I was involved in a cardiac arrest where a family member present was very loud, shouting at staff to call in this doctor and that doctor and pacing back and forth in the room constantly. This was extremely disruptive and increased the risk of miscommunication, which, in turn, could lead to incorrect treatment’*.

Communicating effectively with vulnerable family members and preparing them for what they might witness in the resuscitation room were identified as significant challenges. Participants stressed that family-witnessed resuscitation decisions must be context-dependent, considering factors such as the family member’s relationship with the patient, emotional and cognitive capacity, and understanding of the situation. As one physician noted, *‘It’s not appropriate to establish a standardized policy regarding whether family members should or should not be present. This decision needs to be assessed based on the specific circumstance’.*

Children and adolescents posed a particular ethical and practical dilemma. Participants expressed concerns that witnessing resuscitation could be too traumatic for young individuals, who may lack the emotional maturity to process the experience. Determining an appropriate age threshold for presence in the resuscitation room was a recurrent challenge.

Team discussions were highlighted as essential for managing family-witnessed resuscitation, especially in cases involving vulnerable family members. Collaborative discussions enabled staff to navigate complex dynamics and ethical challenges. Incorporating family perspectives into cardiopulmonary resuscitation training, such as through role-playing scenarios, was also seen as beneficial. A nurse emphasised, *‘I think it's very important in cardiopulmonary resuscitation training to talk about who should be in the room during a cardiac arrest. Then we can capture everyone's thoughts, fears, and opinions in the work group and work together also on ethical issues’.*

Finally, participants also emphasised alternative approaches for situations where family members either chose not to be present or were deemed unsuitable to remain in the room during resuscitation. These included allowing family members to stay just outside the room with partial visibility or waiting nearby while receiving regular updates. Maintaining a sense of control for the family was seen as critical, enabling them to decide whether to stay, leave, or return. As one nurse stated, *‘It would never happen that family members were not allowed to attend cardiopulmonary resuscitation at my workplace. If they don't want to be there, they obviously don't have to be, but everyone who wants to attend can. If you don't have staff who can be allocated to be there and inform family members, it's not ideal, but that would never be a reason for not inviting them to attend. They are always welcome to stay, and they can go in and out of the room if they wish’.*

#### 3.2.2 Working under pressure

Working under pressure encompassed experiences and attitudes related to navigating operational constraints during family-witnessed resuscitation, such as limited room capacity and the risk of breaching confidentiality. Additional concerns included limited family support and disruptive family behaviours that could impair focus during resuscitation. Of the three identified categories, 39 % of the reported experiences and attitudes were associated with this second category.

##### 3.2.2.1 Navigating operational constraints

The participants highlighted several operational challenges that could hinder family presence during resuscitation. One challenge identified was the potential breach of confidentiality during cardiopulmonary resuscitation, particularly regarding sensitive patient information. A physician noted, *‘There may be a breach of confidentiality depending on what needs to be communicated about the patient in the room’.* Concerns were also raised about managing large numbers of family members. Staffing shortages, especially during night shifts, could prevent adequate support for family members, further complicating the decision to offer them presence during resuscitation. One nurse reflected, *‘When this happens at night, there are too few staff present to support the family during resuscitation attempts’.*

In some cases, rooms were too small to accommodate family members, so they were invited to observe from the doorway, ensuring they did not obstruct the team. As one nurse explained, *‘Family members can always stay at a distance, with a good view of the patient and the team, but at a ‘safe distance’ to avoid being in the way. When there has been space, they have been welcome to touch and speak to the patient’.* Nearly half of the nurses expressed concerns that overcrowding could impair the resuscitation team's effectiveness. Examples were given when family members wished to be too close to the patient, which created challenges for the team. Despite concerns, the risks associated with family presence were often manageable, and the presence of family was also seen to have a calming effect. One nurse reflected, *‘The atmosphere in the room is usually calmer when family members are present. We don’t work better or worse, but the work during resuscitation becomes more efficient. It’s not about trying harder to resuscitate the patient, but it’s calmer and more structured, making it easier for everyone to work. In addition to saving the patient, you want to provide the family with a positive memory from the traumatic situation, and it feels more respectful, giving them a clearer understanding of the situation.*

##### 3.2.2.2 Limited family support

Many participants supported family-witnessed resuscitation, provided there was sufficient staff available to support the family members. This support included answering their questions, explaining the procedures, and providing reassurance about what was happening. However, challenges arose when there were insufficient personnel to manage both the resuscitation process and family support. As one nurse explained, *‘If there is enough staff for one person to be with the family member, that is ideal. But sometimes, we are the only ones needed to maintain effective cardiopulmonary resuscitation, and, in those cases, it can be difficult to allocate a staff member to the family member. Because I think it's necessary to have someone present with them, so that they don’t feel abandoned, frightened, and without support’.* The participants also expressed concerns that, in such situations, family members might be sidelined, without adequate explanation of what is happening.

Other complicating factors if a dedicated family support person was lacking included family members who were disruptive or emotionally overwhelmed. One nurse highlighted the tension between patient care and family support, *‘There’s no time for family members during an acute cardiopulmonary resuscitation situation, where all focus must be on the person being resuscitated. Diverting attention to family members with the limited resources available would reduce the effectiveness of the work and thus our ability to succeed in resuscitation’.*

However, most participants did not consider the lack of family support to be a valid reason to deny family presence. In such instances, participants suggested that family members could be informed about the staffing limitations. Furthermore, it was acknowledged that some family members might prefer to leave the room, and this decision needed to be respected by support provided in another location. While most situations were manageable with the available staff, many participants expressed a desire for further training in managing family members during and after cardiopulmonary resuscitation. As one nurse stated, *‘During the last cardiopulmonary resuscitation training exercise, we practised with family members present, which was very instructive’.*

Finally, several participants also highlighted the importance of follow-up care for family members immediately after a cardiac arrest, suggesting that a range of professionals, such as nurses, physicians, and social workers, should be involved. Many physicians, however, felt it was their responsibility to provide information to the family. As one physician explained, *‘I’m usually the one leading the team, so I don’t have time to explain or be present for the family during the cardiopulmonary resuscitation process. However, I always make sure to meet with the family afterwards and have a conversation with them’.*

##### 3.2.2.3 Disruptions from family behaviour and impaired focus

The participants expressed concerns about feeling under "critical surveillance" during resuscitation efforts, a scenario perceived as stressful regardless of family presence. Some described discomfort from being observed by family members, fearing it could impair their focus and negatively impact the team’s performance. There were concerns that family members might challenge medical decisions, which could lead to unnecessary discussions in high-pressure situations. Participants working in units where resuscitation was less frequent reported heightened anxiety and insecurity when family members were present, *‘I think staff can be distracted when family members are present. Cardiac arrest situations are often chaotic, and having family in the room can make it more challenging for staff, particularly in departments like mine, where cardiac arrests are rare, and we are less experienced* [nurse]*’.*

A widespread fear was that family members could disrupt the resuscitation by being physically intrusive, emotionally volatile, or even aggressive, which could divert attention from the patient. Such behaviour was viewed as a potential barrier to effective teamwork and communication as described by this nurse, *‘Having a family member near the patient during cardiac arrest could hinder staff performance, both physically and mentally. If the family member becomes loudly distressed or hysterical, it disrupts focus and communication among the team. Highly emotional or outwardly reactive behaviour can significantly impair our ability to concentrate’.*

Another concern was that family members might perceive the situation as chaotic and disorganised, misinterpreting medical jargon and procedures. The participants worried that this could result in blame or accusations if the outcome was unfavourable. Misunderstandings or distrust of the medical team, particularly when resuscitation was unsuccessful, were also sources of anxiety as reflected by this physician, *‘The family is an additional factor to manage emotionally. In a complex resuscitation where I’m leading the process, I don’t want to adapt to yet another variable. I fear that my decisions may be influenced or that I may need to justify certain actions – or the lack thereof’.*

Complicated family dynamics or pre-existing tensions between the staff and the family were factors that could further hinder family-witnessed resuscitation as described by this physician, *‘*Challenges include prior conflicts between staff and family, disagreements over treatment goals, or complex family situations’. Language barriers and cultural differences were also highlighted as obstacles. Unfamiliar expressions of grief or difficulty explaining medical procedures due to linguistic challenges could create discomfort among staff. In some cases, cultural misunderstandings escalated tensions, particularly when families struggled to accept the decision to discontinue resuscitation efforts as exemplified by this nurse, *‘*I experienced a failed resuscitation attempt where the family reacted very strongly - loud screams, violent sobbing, and continuous chanting throughout the process. I felt that this affected the team’s focus, and the resuscitation was prolonged beyond what felt ethically defensible, likely influenced by the families reactions’.

Finally, some participants cited safety concerns, including threats, violence, or the presence of individuals with criminal backgrounds, as reasons to exclude family members during resuscitation. Rare cases were also described where a family member actively tried to sabotage the resuscitation, such as when the family member had previously harmed the patient and did not want the resuscitation to succeed.

#### 3.2.3 Helping or harming the family

Helping or harming the family captured the dual perception that family presence could facilitate understanding of the resuscitation process and support grieving if the patient did not survive, while also posing a risk of traumatic experiences for family members. Of the three identified categories, 49 % of the reported experiences and attitudes were associated with this third category.

##### 3.2.3.1 Family presence facilitates understanding and grieving

Many participants recognised the value of involving family members during resuscitation, as it was seen to benefit both the patient and their family. When family members stayed close to the patient, participants could explain the resuscitation process in real-time and address their questions, which fostered understanding and acceptance of the rationale behind decision-making during resuscitation. It enabled family members to comprehend the resuscitation team’s actions and reassures them that everything possible has been done. As one physician stated, *‘Family members are less likely to have a distorted view of the medical interventions if they are present during resuscitation. They are more likely to understand why certain decisions are made’*.

Many participants believed that witnessing resuscitation helped the family process the patient’s final moments, alleviating doubts about whether more could have been done. They noted that family members often imagined scenarios during resuscitation that were far worse than the reality. As reflected by this nurse, *‘I believe that it can feel good afterwards for the family member to know that you were there and with the patient to the end. So, it's for the family member's sake above all that they should be given the chance to be present. As a family member, you are at the mercy of your loved one, unable to help them. If you can at least be there, feel that you have been close and not ‘abandoned’ your loved one in what may be the last moment of life, I believe that it can provide comfort and a sense of meaning for the family member to carry with them through the grief’.*

The opportunity to stay with a loved one until the very end, rather than losing precious time, was described as deeply significant for family members and crucial in easing the grieving process. The family could feel that they were part of the final moments and perhaps have the opportunity to say goodbye in a way they otherwise might not have been able to, *‘Over my many years in the emergency room, I have had numerous family members present during cardiopulmonary resuscitation situations. All have been grateful for the chance to witness the resuscitation, regardless if the patient survived or died. One woman whose husband did not survive told me, ‘I didn't realise what you were doing to my husband, but I will be forever grateful that I was there [nurse]’.*

Another nurse reflected, *‘After all, perhaps it is enough to be human – to consider the pain of being excluded from the final moments of the lives of those we love most’.* Both physicians and nurses recognised that family-witnessed resuscitation helped families process the event, irrespective of the outcome of cardiopulmonary resuscitation. Being present during resuscitation was seen as a way to help family members understand what happened to the patient and provide support during a crisis. As one physician stated, *’family-witnessed resuscitation helps the family member continue grieving. My belief is that it can make the situation feel real in a completely different way, providing certainty that the death was inevitable’.*

Finally, both physicians and nurses highlighted that results from several research studies supported the positive outcomes of family-witnessed resuscitation. They noted that researchers have shown that presence of family members during cardiopulmonary resuscitation leads to a better understanding of the process and reassurance that everything was done to save the patient. However, there were also a few participants expressing scepticism, as described by this physician, *‘I don't believe that all family members would feel better by attending and witnessing a cardiopulmonary resuscitation situation, but maybe some’.*

##### 3.2.3.2 Traumatic sensory impressions

A common concern among the participants was that witnessing resuscitation could be excessively traumatic for family members, exposing them to distressing and graphic scenes. Extreme resuscitation efforts were described as resembling a "horror film," particularly in cases involving severely injured patients, such as those involved in major accidents with extensive external injuries or when mechanical chest compressions are given. One nurse stated, *‘Resuscitation can take place when the room is very messy, as for example severe bloody vomiting during cardiopulmonary resuscitation, and there is no one available to care for family members. Sometimes the circumstances are far from pleasant to witness’.* The participants emphasised the importance of preparing family members before allowing them to witness resuscitation, including explaining the potentially-distressing aspects, such as the forceful nature of chest compressions, the sound of ribs breaking, or the sights and sounds associated with defibrillation as described by this nurse, *‘A resuscitation attempt can add another layer of trauma for family members, such as hearing ribs crack, witnessing intraosseous injections, seeing the body's reactions to defibrillation, or hearing the noise from the defibrillator. Without preparation, these experiences can be overwhelming and deeply distressing’.* Some participants expressed concern that witnessing resuscitation could have lasting psychological effects on family members, including intrusive memories or symptoms of post-traumatic stress. They emphasised the need to consider whether such potentially-distressing experiences might become the family’s final memory of their loved one and expressed uncertainty about how family members might wish to remember their loved one. This uncertainty could influence the decision to invite them to witness the resuscitation.

Finally, the participants also expressed concerns about being perceived as lacking empathy due to the intense and clinical nature of resuscitation efforts, which can appear stark and impersonal as reflected by this nurse, *‘Resuscitation is not a graceful process. It’s systematic and highly goal-oriented, and if family members are unfamiliar with the procedures, it can seem quite macabre’.*

## 4 Discussion

We have added new insights to the limited qualitative research available on how experiences and attitudes influence healthcare professionals’ actions regarding family-witnessed resuscitation across various care settings and hospital levels. We have emphasized the importance of balancing legal, ethical, and practical considerations while balancing the needs of both patients, families, and resuscitation staff in family-witnessed resuscitation situations. The main findings were that participants were either committed to or undecided about family-witnessed resuscitation, depending on the context. This underscores the complexity of family-witnessed resuscitation and highlights the challenges associated with its implementation in clinical practice. A unique contribution of the present study is the recognition of 'fragile individuals' in the context of family-witnessed resuscitation, a topic sparsely addressed in previous research.

A key concern among participants was the potential for traumatic experiences among family members witnessing resuscitation, fearing that graphic scenes could result in lasting distress. This hesitation aligns with existing literature (Grimes, 2020; Tíscar-González et al., 2021). Nevertheless, researchers have suggested that most families who experience an in-hospital resuscitation of a loved one would choose to be present again (Considine et al., 2022), indicating that the opportunity to witness the efforts to save the patient and remain close during such critical moments outweighs the distress. Our findings echo this benefit, as most participants emphasised that family-witnessed resuscitation supported families’ sense of participation and closure. The participants considered it important for families to be offered the opportunity to be present during cardiopulmonary resuscitation, as this allowed them to remain close to the dying patient, receive explanations about the ongoing resuscitation process, and ask questions. The participants also highlighted that family members could serve as a source of support and hope for the patient, a perspective previously described as significant (Barreto et al., 2019; Tennyson, 2019). If the patient did not survive, participants emphasized that family-witnessed resuscitation could facilitate the family’s processing of the event. This benefit has also been described as one of the positive aspects of family-witnessed resuscitation (Vardanjani et al., 2021). Another reason for the participants to offer family-witnessed resuscitation was its potential benefit for the patient in the event of survival. In such cases, family members were seen as a valuable source of support during recovery, as they could recount the events to the patient, aiding in their processing and rehabilitation. This perspective has also been confirmed by cardiac arrest survivors (Waldemar et al., 2023).

The participants also expressed apprehension about being critically observed, feeling scrutinized, and fearing negative impacts on team dynamics and resuscitation outcomes in the presence of family members. Similar concerns are well-documented (Tennyson, 2019; Tíscar-González et al., 2021). However, researchers have found no adverse effects on resuscitation processes or outcomes (Goldberger et al., 2015; Oczkowski et al., 2015; Vardanjani et al., 2021; Waldemar et al., 2021). Interestingly, some participants described family-witnessed resuscitation as promoting more structured and empathetic care, emphasizing the importance of creating a positive memory for the family in a challenging situation. These contrasting perspectives underline the duality of family-witnessed resuscitation experiences, where the presence of family members can evoke both discomfort and improved team composure.

Challenges also included the lack of knowledge about patients’ preferences for family-witnessed resuscitation, raising ethical dilemmas related to autonomy, confidentiality, and integrity, as previously discussed (Grimes, 2020; Tíscar-González et al., 2021). Engaging in discussions about fundamental ethical principles, such as beneficence, was considered valuable by the participants. They also highlighted logistical challenges, including limited space and distractions during cardiopulmonary resuscitation. These issues are commonly reported (Vardanjani et al., 2021). To address them, many participants proposed solutions, such as allowing families to observe from a doorway or other non-obstructive positions. They emphasised the importance of preparing families for the resuscitation environment, which could otherwise be perceived as ‘messy’ due to the intensity of the work and the number of people involved. Pre-resuscitation conversations to set expectations were considered essential and align with existing recommendations (Tennyson et al., 2023; Toy, 2023).

Previous researchers have demonstrated the value of physical contact during family-witnessed resuscitation for both family and patient, highlighting the importance of allowing family members to touch or hold the patient at some point during resuscitation (Barreto et al., 2019; Toy, 2023). However, some participants in the present study expressed scepticism due to concerns about interference with resuscitation efforts. Nonetheless, the participants also acknowledged that family members could be allowed to approach, touch, or speak to the patient when appropriate, suggesting that such interactions can often be resolved.

Safety risks, such as the influence of drugs or alcohol and disruptive behaviours with unwillingness to follow instructions, were also stressed in our study, issues that have received limited attention in previous research. While rare, instances of aggression or conflict with medical teams (Jabre et al., 2013) emphasize the importance of addressing these concerns in family-witnessed resuscitation training, particularly in the increasingly multicultural composition of modern societies characterized by language barriers and cultural or traditional differences. In the present study, concerns were raised about fears of threats, violence, and interference from individuals with criminal backgrounds during resuscitation. These concerns warrant serious consideration, as workplace violence in healthcare settings is an escalating global issue (O'Brien et al., 2024). For example, a study from Iran highlighted the prevalence of violence against nurses during cardiopulmonary resuscitation attempts in the emergency department as a significant issue (Margavi et al., 2020). Approximately 20 % of healthcare professionals reported having experienced physical abuse during their working shift (Li et al., 2020), which could severely impact their physical and psychological well-being, leading to depression, reduced self-esteem, sleep disturbances, and symptoms of post-traumatic stress disorder. Such violence not only harms healthcare professionals but also may compromise the quality of care delivered (Lim et al., 2022). These findings underscore the urgent need to increase awareness and implement measures to prevent workplace violence in healthcare. Failure to address this issue could negatively affect staff well-being and, in extreme cases, impair the delivery of life-saving interventions, such as cardiopulmonary resuscitation, potentially reducing healthcare professionals’ willingness to involve family members during resuscitation. Hence, family-witnessed resuscitation training sessions should address managing diverse dynamics while balancing legal and ethical responsibilities (Toy, 2023; Vardanjani et al., 2021).

Lastly, scarce human resources emerged as a significant barrier for participants to support family members during resuscitation, echoing previous researchers (Vardanjani et al., 2021). However, numerous researchers have described efforts to address this issue by involving other designated resources, such as chaplains, counsellors, and anaesthesia technicians, to support families during and after resuscitation (Cornell and Powers, 2022; Jabre et al., 2013; James et al., 2011; Powers et al., 2023). Establishing clear local routines and guidelines is crucial to ensure ethical, safe, and effective family-witnessed resuscitation implementation. Whether support is provided by external resources or by existing staff allocated within the unit, such practices are essential to standardise the process and support families effectively during resuscitation. Based on our findings, we have suggested key areas for targeted improvements and practical clinical applications for the healthcare environment studied, described in Table 3.

**Table 3:** Suggestions for clinical implications and actions, based on key findings.

Findings	Clinical Implications	Actions
Respecting patient and family wishes	- Ensuring family-centred care aligns with ethical and patient autonomy principles	- Clarify patient and families preferences regarding family-witnessed resuscitation. - Conduct team discussions, skill training, and role modelling to prepare staff for ethical and practical considerations in family-witnessed resuscitation. - Develop local policies for managing family-witnessed resuscitation based on the circumstances of each unique unit.
Challenges posed by vulnerable family members	- Balancing support for families with maintaining resuscitation focus. - Addressing distress among families.	- Train staff in managing vulnerable family dynamics. - Assign existing staff or external resources to support families.
Family presence facilitates understanding and grieving	- Recognizing the role of family-witnessed resuscitation in fostering closure and reducing long-term grief. - Supporting family participation in critical moments.	- Provide real-time explanations to family members during resuscitation when possible. - Offer debriefing sessions after resuscitation to address questions and provide emotional support.
Navigating operational constraints	- Ensuring safe and effective cardiopulmonary resuscitation while accommodating family-witnessed resuscitation. - Addressing logistical challenges to optimize team performance. - Addressing confidentiality risks to ensure patient and family privacy during family-witnessed resuscitation. - Balancing the need for transparency with the ethical obligation to maintain patient dignity.	- Designate observation areas for families. - Ensure staff are trained to assess and adapt to the physical environment during cardiopulmonary resuscitation. - Assign a specific team member to support and manage family presence in real-time during resuscitation. - Develop strategies to shield sensitive information (e.g., screens or physical barriers) when feasible during resuscitation. - Train staff to communicate clearly about confidentiality and appropriate boundaries with family members present during family-witnessed resuscitation. - Develop local policies within each healthcare unit to address the practical aspects of family-witnessed resuscitation, including how healthcare professionals can ensure integrity, communicate empathically, assess preferences, incorporate values and collaborate with other disciplines ensuring that healthcare professionals feel confident and supported in these situations.
Limited family support	- Highlighting the need for consistent resources to support families during family-witnessed resuscitation. - Recognizing the burden of insufficient human resources.	- Allocate external resources such as counsellors or chaplains. - Provide staff training in family communication.
Disruptions from family behaviour and impaired focus	- Mitigating risks of distractions or interference during cardiopulmonary resuscitation. - Balancing ethical and safety considerations in the presence of disruptive behaviours.	- Establish protocols for handling disruptive family members. - Include conflict management in family-witnessed resuscitation training. - Hospital managers must recognize and address the escalating workplace violence in healthcare, ensuring measures are in place to protect healthcare professionals performing cardiopulmonary resuscitation and support the safe implementation of family-witnessed resuscitation.
Traumatic sensory impressions	- Minimizing potential trauma for families exposed to graphic resuscitation scenes. - Preparing families to reduce distress and improve understanding of resuscitation efforts.	- Provide pre-resuscitation briefings for families. - Designate support personnel to guide and comfort families during family-witnessed resuscitation. - Offer real-time explanations to families about what is happening in a calm and clear manner to provide context and reduce shock. - Provide post-resuscitation debriefings to help families process the event and offer psychological support if needed.

### 4.1 Strengths and limitations

There are some potential limitations to consider when interpreting the findings of this study, as they may influence its trustworthiness. One challenge of using open-ended questions in qualitative research, where data is neither systematically nor purposively collected, is the lack of structure in the material. This can complicate the data analysis process and may limit the depth of insights gained. The representativeness of the participants in relation to the research question is also a potential concern. A significant proportion of the participants worked in emergency and medical departments, likely providing them with more experience of family-witnessed resuscitation compared to participants from other units. This uneven distribution may have influenced the findings. Another limitation is that some participants in the intervention study did not answer the open-ended questions, likely due to time constraints. Providing additional time for survey completion might have mitigated this issue. Furthermore, those who chose to provide written comments may have been more articulate or held stronger opinions – either critical or positive – about family-witnessed resuscitation than the average respondent. Finally, approximately half of the participants had direct experience of family-witnessed resuscitation, while the others based their reflections on assumptions, beliefs, and attitudes rather than lived experience. However, whether rooted in direct experience or preconceptions, healthcare professionals’ attitudes inevitably influence their decisions on whether to invite family members to be present during resuscitation. It is therefore essential to study both experienced-based attitudes and preconceived notions, as both contribute to shaping clinical practice. Given these considerations, readers should focus on the transferability of the findings to similar contexts, rather than their generalizability.

Despite its limitations, this study also has several notable strengths. A rigorous summative content analysis was conducted, with both authors collaboratively discussing codes and categories. This approach enhanced the credibility of the findings and ensured methodological consistency throughout the research process. Although the representativeness of the respondents can be questioned, it is noteworthy that 75 % of the participants in the educational intervention provided free-text comments. There were no significant demographic differences between those who responded and those who did not. This strengthens the argument that the findings offer valuable insights into well-established aspects of family-witnessed resuscitation. Moreover, from our findings, we reinforced previous research while also highlighting new perspectives. The inclusion of free-text responses added depth to the analysis, offering a more nuanced understanding of family-witnessed resuscitation compared to relying solely on closed-ended questions. These insights contribute additional knowledge that healthcare systems can draw upon to inform discussions and decision-making around family-witnessed resuscitation.

### 4.2 Conclusions

We have highlighted the multifaceted nature of family involvement during resuscitation, encompassing their presence, participation as caregivers and recipients, and involvement in decision-making processes. Family-witnessed resuscitation presents significant ethical and practical challenges for healthcare professionals. A lack of understanding among healthcare professionals regarding family-witnessed resuscitation and the roles of family members during such events can lead to resistance to its implementation. To address this, healthcare professionals could engage in discussions about fundamental ethical principles – beneficence, non-maleficence, autonomy, and justice – from the perspectives of patients, families, and healthcare teams. Encouraging open dialogue and respecting the wishes of patients and their families are essential steps in aligning care practices with these principles. Despite evidence supporting the benefits of allowing family members to have physical contact with the patient during resuscitation, hesitation among some healthcare professionals persists, underscoring the need for targeted education and training. Additionally, strategies could be developed to address the unique needs of vulnerable individuals, minors, and families from diverse cultural and religious background in family-witnessed resuscitation situations. Finally, the concern among healthcare professionals about threats and violence during resuscitation warrants attention, as workplace violence in healthcare settings continues to rise globally.

## Declaration of generative AI in the writing process

During the preparation of this work, the authors used ChatGPT in order to edit language and readability of the text in some parts. This was followed by the authors reviewing and editing the content as needed. The authors take full responsibility for the content of the published article. No participant quotes has been entered into ChatGPT.

## CRediT authorship contribution statement

**Annette Waldemar:** Writing – review & editing, Writing – original draft, Visualization, Validation, Resources, Project administration, Methodology, Investigation, Formal analysis, Conceptualization. **Ingela Thylén:** Writing – review & editing, Writing – original draft, Validation, Supervision, Software, Methodology, Investigation, Funding acquisition, Formal analysis, Data curation, Conceptualization.

## Declaration of competing interest

The authors declare the following financial interests/personal relationships which may be considered as potential competing interests:

Ingela Thylen reports financial support was provided by the Medical Research Council of Southeast Sweden (grant number FORSS-980620). If there are other authors, they declare that they have no known competing financial interests or personal relationships that could have appeared to influence the work reported in this paper.

## Data Availability

The datasets generated or analysed during the current study are not publicly available but will be available from the corresponding author upon reasonable request.
